# The synergistic effects of zinc oxide nanoparticles and fennel essential oil on physicochemical, mechanical, and antibacterial properties of potato starch films

**DOI:** 10.1002/fsn3.2371

**Published:** 2021-06-01

**Authors:** Hamid Babapour, Hossein Jalali, Abdorreza Mohammadi Nafchi

**Affiliations:** ^1^ Food Biopolymer Research Group Food Science and Technology Department Islamic Azad University Damghan Iran; ^2^ Food Technology Division School of Industrial Technology Universiti Sains Malaysia Penang Malaysia

**Keywords:** active packaging, bionanocomposite film, essential oil, nanotechnology

## Abstract

The purpose of this study was to evaluate the effects of a combination of zinc oxide (ZnO‐N) nanoparticles and fennel essential oil (FEO) on the functional and antimicrobial properties of potato starch films. Films based on potato starch containing a combination of ZnO‐N (1, 3, and 5%(w/w)) and FEO (1, 2, and 3% (w/w)) produced by casting method and water solubility, water absorption capacity (WAC), barrier properties, mechanical properties, color indexes, and antimicrobial activity of the films against *Staphylococcus aureus*, *Escherichia coli,* and *Aspergillus flavus* were studied. The combination of ZnO‐N and FEO had a significant decreasing effect on solubility, WAC, water vapor and oxygen permeability, elongation, and L* index. These additives had an increasing impact on tensile strength, Yang's modulus, and a* and b* indexes (*p* < .05). By increasing the concentration of ZnO‐N and FEO, the antimicrobial activities of bionanocomposite films significantly increased (*p* < .05). Both ZnO‐N and FEO had a significant effect in this respect, although the effects of ZnO‐N were more significant. In conclusion, an excellent synergistic effect of ZnO‐N and FEO was observed in potato starch films.

## Graphical abstract

Active bionanocomposite starch film with zinc oxide nanoparticles and fennel essential oil was prepared. By increasing the concentration of ZnO‐N and FEO, the antimicrobial activities of bionanocomposite films significantly increased. An excellent synergistic effect of ZnO‐N and FEO was observed in potato starch films.

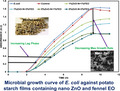

## 1 INTRODUCTION

Today, the demand for packaging films based on biopolymers has increased because, unlike synthetic polymers, they are environmentally friendly (Toscano Ávila et al., 2020; Xue Mei et al., 2020). These biopolymers include polysaccharides, proteins, and lipids (Islam et al., 2020; Jahdkaran et al., 2021). Starches are one of the most important biopolymers used to make biocomposites. They are nontoxic, available, biodegradable, and renewable and have low prices (Esfahani et al., 2020). Potato starch contains high amounts of amylopectin, and its granule size is large compared to other starches. This starch offers several advantages in paste clarity, transparency, and extensibility (Alcázar‐Alay & Meireles, 2015; Aminian et al., 2013).

Since biopolymer‐based films usually have poor barrier and mechanical properties, to overcome these problems, either these polymers are used in combination, or plasticizers, fillers, and/or cross‐linking agents are used in biocomposite films. The use of nanoparticles in packaging films is one of the best ways to improve physicochemical and functional characteristics such as antioxidant and antimicrobial activity (Ahmadi et al., 2019; Jafarzadeh et al., 2021). Metal and metal dioxide nanoparticles are most widely used in packaging films due to their optical characteristics, good flexibility, impermeability to gases, and antimicrobial activity (Jafarian et al., 2020).

ZnO is a metal nanoparticle with antimicrobial activity approved by the Food and Drug Administration (FDA) (Li et al., 2019). These nanoparticles have good biocompatibility and thermal stability and are nontoxic (Huang et al., 2017). Researchers have shown that ZnO nanoparticles show strong and significant antibacterial activity against both gram‐positive and gram‐negative bacteria (Shahvalizadeh et al., 2021).

Plant essential oils (EOs) extracted from different parts of plants have a complex combination of various active and aromatic compounds (Chang et al., 2021; Plant et al., 2019). EOs often demonstrate significant antimicrobial and antioxidant activities and can be used as a substitute for synthetic preservatives in the food industry (Moslehi et al., 2021; Mousavian et al., 2021). Sun et al. (2020), in an investigation of the effect of ZnO nanoparticles and mulberry extract on the chitosan/konjac glucomannan films, demonstrated that the use of these additives in film samples improves barrier, mechanical, thermal stability, optical properties, and antimicrobial activity. In another study, it was found that titanium dioxide and *Zataria multiflora* essential oil had synergistic effect on each other and improved the basic and antimicrobial activity of chitosan/whey protein‐based bionanocomposite films (Gohargani et al., 2020).

Fennel, with the scientific name of *Foeniculum vulgare* L., is a plant of the *Apiaceae* family that is widely cultivated all over the world due to its aromatic seeds and leaves. The essential oil of fennel is an important source of medicinal and active compounds such as antioxidants and antimicrobial compounds (Chang, Mohammadi Nafchi, et al., 2016). The antimicrobial activity of fennel essential oil has been reported in different studies (Chang, Abbaspour, et al., 2016; Gonçalves et al., 2020).

There is no report on the effect of ZnO nanoparticles and fennel essential oil on potato starch edible films that is yet available to the best of our knowledge. Therefore, the purpose of this study was to develop an active bionanocomposite film based on starch potato containing a combination of ZnO nanoparticles and fennel essential oil and to investigate the physicochemical, mechanical, and antimicrobial activity of obtained bionanocomposite films.

## 2 MATERIALS AND METHODS

### 2.1 Materials

Potato starch was purchased from SIM Company Bhd (Penang, Malaysia). ZnO nanoparticles and glycerol as a plasticizer were purchased from Sigma Chemical Co. (St. Louis, MO, USA). Fennel essential oil was purchased from Barij‐Essence Co. (Iran). Microbial strains, including *Staphylococcus aureus*, *Escherichia coli* O157:H7, and *Aspergillus flavus,* were prepared from the Organization of Scientific and Industrial Research of Iran. All other chemicals and culture mediums used in this study were of analytical grade and purchased from Merck Co. (Germany).

### 2.2 Preparation of potato starch/ ZnO‐N/fennel essential oil bionanocomposite films

To properly distribute the ZnO nanoparticles in the film structure, these nanoparticles were first dispersed uniformly in water, and then, this water was used to prepare potato starch dispersion. To prepare nanosolutions with concentrations of 1, 3, and 5% (nanoparticle weight to dry weight of starch), first, a suitable amount of ZnO nanoparticles was poured into 100 ml of distilled water. To ensure the homogeneity of the nanosolutions, they were homogenized in an ultrasonic bath (BANDELIN SONOREX Digitec, Germany) for 20 min. Then, 4 g of potato starch was added to the nanosolutions and mixed. After that, 2 g glycerol (50% w/w) was added as a plasticizer and stirred. While stirring, the suspension was heated to 90℃ and kept at this temperature for 45 min to complete gelatinization of the starch. While the mixture was cooling, a suitable amount of fennel essential oil (1, 2, and 3%) was added to the film solution at 45℃ temperature and became homogenous for approximately 30 min. Then, about 90 g of the final mixture was poured on the Plexiglas and placed in a specific oven (Memmert, Germany) at 25℃ and 50% relative humidity for 24 hr to dry the film. Finally, the dried film was separated from Plexiglas and placed desiccator that relative humidity was fixed between 50% and 60% until the films were tested (Akbariazam et al., 2016; Teymourpour et al., 2015).

### 2.3 Determination of the thickness of the films

The thickness of the starch films was measured by a caliper (QLR IP54, America) with an accuracy of 0.001 mm. Measurements were performed at three points on the films, and their average was used in calculations related to physicochemical properties tests such as water vapor permeability.

### 2.4 Determination of water solubility

Initially, pieces of films were cut and stored in a desiccator with calcium chloride (0% relative humidity) and in an oven for 1 day. The samples were then weighted and mixed in a beaker containing 100 ml of deionized water, then covered with aluminum foil, and placed at room temperature (20–25℃) for 24 hr. Meanwhile, the samples were gently stirred every 4 hr for 10 min. The remaining pieces of films were filtered with filter paper and placed in an oven at 30℃ to stabilize the weight. Finally, the water solubility percentage of the films was calculated through Equation (1) (Ekramian et al., 2021a):

(1) $$\text{Water solubility}\;\left(\%\right)\;=\frac{w_{2}‐w_{1}}{w_{2}}\times 100.$$

where *w*_1_ was the dry film weight (g), and *w*_2_ was the swollen film weight (g).

### 2.5 Determination of water absorption capacity (WAC)

To determine the water capacity of the films, pieces of film were first cut and placed in a desiccator with calcium chloride and kept in an oven at 30℃ for one day. The film samples were then stored in a desiccator containing deionized water for 24 hr. After that, the film pieces were removed from the desiccator and weighted again. Finally, the water absorption capacity of the films was obtained through Equation (2) (Torabi & Mohammadi Nafchi, 2013):

(2) $$\text{WAC}\;\left(g/\text{g dried film}\right)\;=\frac{\text{weight of absorbed water}}{\text{dry weight of the film}}$$

### 2.6 Determination of water vapor permeability (WVP)

The WVP amounts of the bionanocomposite films were examined following ASTM Standard E96/E96 M‐16 (ASTM, 2016) with some modifications (Ekramian et al., 2021b). The standard glass cups were filled with activated silica gel 1 cm below the film surface. The film thickness was measured and fitted to the cups and put in a desiccator containing saturated magnesium nitrate to provide 55 ± 2% RH at 25℃. The weights of the cups were measured at certain times during the 7 days. Then, the diagram of changes in water moisture content was drawn, and the slop of the line was used to calculate the water vapor transmission rate (WVTR) (g/day). The WVP amounts of the film samples were estimated by multiplying the steady‐state WVTR by film thickness and dividing that by film area and the water vapor pressure difference inside and outside of the cups (Alipoormazandarani et al., 2015).

### 2.7 Determination of oxygen permeability (OP)

In order to evaluate the oxygen permeability of the film samples, ASTM Standard Method D3985‐17 (ASTM, 2017) and a Mocon Oxtran 2/21 system (Minneapolis, USA) were used. Samples were conditioned at 55% relative humidity and 25℃ temperature for 48 hr and then were measured the thickness and mounted into the diffusion cell of the equipment. Using the convergent method, OP of the films was estimated by WinPermTM permeability software (Abedinia et al., 2018).

### 2.8 Determination of mechanical properties

The mechanical properties of film samples were investigated according to the ASTM‐D882‐18 standard (ASTM, 2018) using a texture analyzer (LLOYD, RS 232, America). The mechanical properties of the films by tensile test (5 kg cell bar and seperation speed of 50 mm/min) were evaluated. For each film, three samples with 150 mm × 50 mm were analyzed. The mechanical parameters measured in this study included tensile strength (TS), Young's modulus (YM), and elongation at break (EB).

### 2.9 Measurement of color parameters

Color of starch film samples was analyzed using a Hunter lab (ColorFlex, America). Color parameters including L* (lightness), a* (redness/greenness), and b* (yellowness/blueness) were studied.

### 2.10 Determination of antimicrobial activity

#### 2.10.1 Agar diffusion method (Static test)

The film samples were cut into 5 mm diameter disks, and the obtained disks were placed on brain–heart infusion medium under sterile conditions. Before placing the disks on the surface of the culture medium, surface culture was performed using 0.1 ml of liquid culture of each of the tested microorganisms (10^5^–10^6^ CFU/ml) such as *E. coli*, S. *aureus,* and *A. flavus*. The plates were then incubated at 37℃ for 24 hr. Finally, the inhibition zones area was measured using a caliper with an accuracy of 0.02 mm (Alebooyeh et al., 2012).

#### 2.10.2 Shake flask method (dynamic test)

The antimicrobial activity of film samples was also studied according to the shake flask method. In this method, bacteria and mold grown in 100 ml of Mueller Hinton Broth were used. 2 × 10^5^ CFU/ml of each microorganism were added to 100 ml of Mueller Hinton Broth medium and incubated at 37℃ for 12 hr. Every 2 hr, the absorbance was recorded by spectrophotometer UV/Visible (Shimadzu, model UV‐1700, Japan) at a wavelength of 600 nm, and microbial growth was obtained. The test was repeated 3 times, and the average values were used (Sadeghnejad et al., 2014).

### 2.11 Statistical analysis

Using the SPSS 22.0 software, the mean of each tested parameter was analyzed by one‐way analysis of variance (ANOVA). Differences between treatments were assessed using Duncan's multiple range test at a 5% level of probability (*p* <.05).

## 3 RESULTS AND DISCUSSION

### 3.1 Effect of ZnO‐N and FEO on the thickness of starch films

**FIGURE 1:**
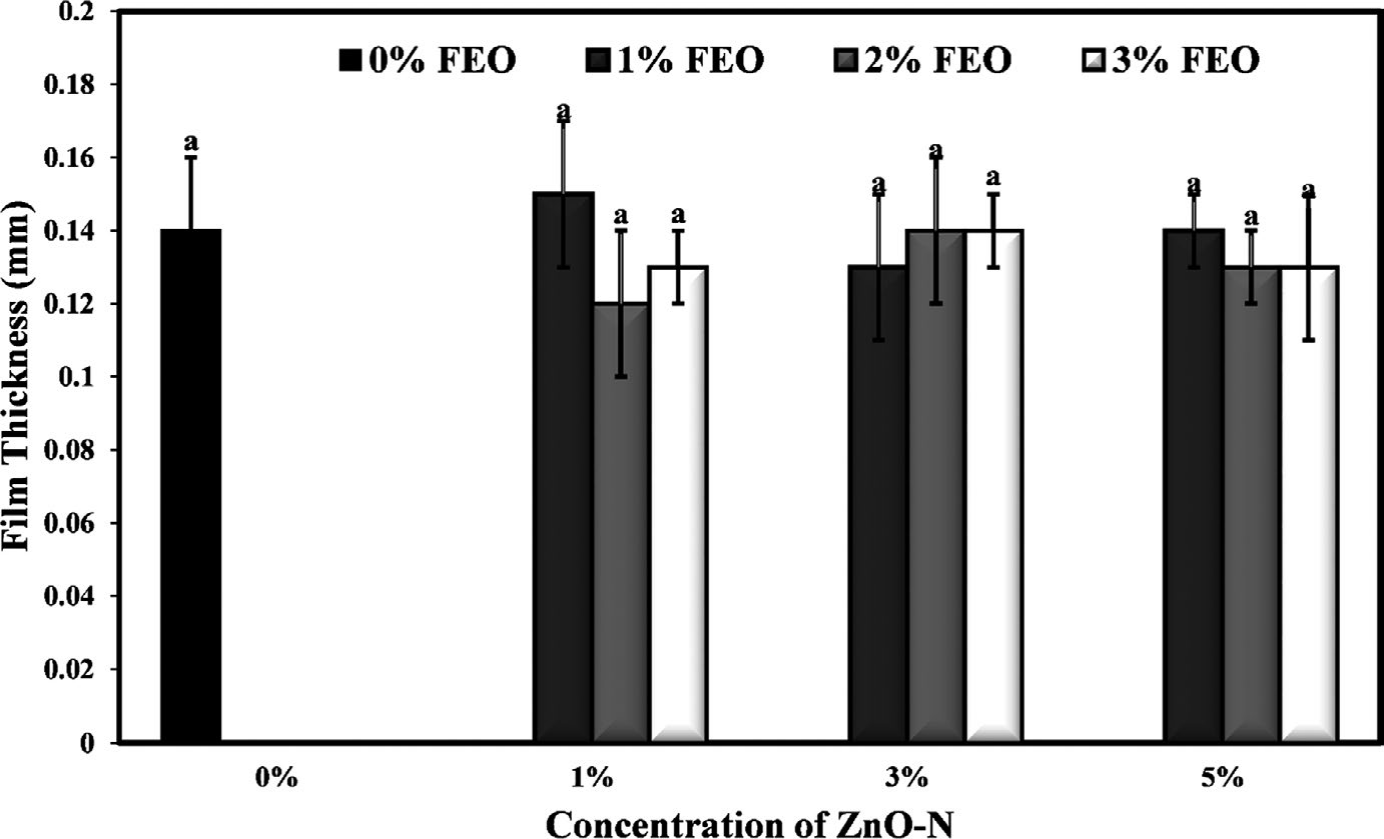
Effects of nano‐ZnO and fennel essential oil on thickness (mm) of potato starch films

The thickness values of the bionanocomposite films containing a combination of ZnO‐N and FEO are compared in Figure 1. They show no significant difference between the mean values of the thickness of starch film samples. The average amount of film thickness was in the range of 0.12–0.15 mm. Sokhtezari et al. (2017) agreed that the addition of *Scrophularia striata* extract to cellulose film had no significant effect on film thickness. Heydari‐Majd et al. (2020) also observed that the thickness of the polylactic acid films did not change significantly by incorporating ZnO nanoparticles and *Zataria multiflora* L. essential oil into film samples. Another study demonstrated that the addition of Miswak root extract and cellulose nanofiber to CMC films increased the thickness of the film samples, but this increase was not statistically significant (Ahmadi et al., 2019).

### 3.2 Effects of ZnO‐N and FEO on the solubility of starch films

**FIGURE 2:**
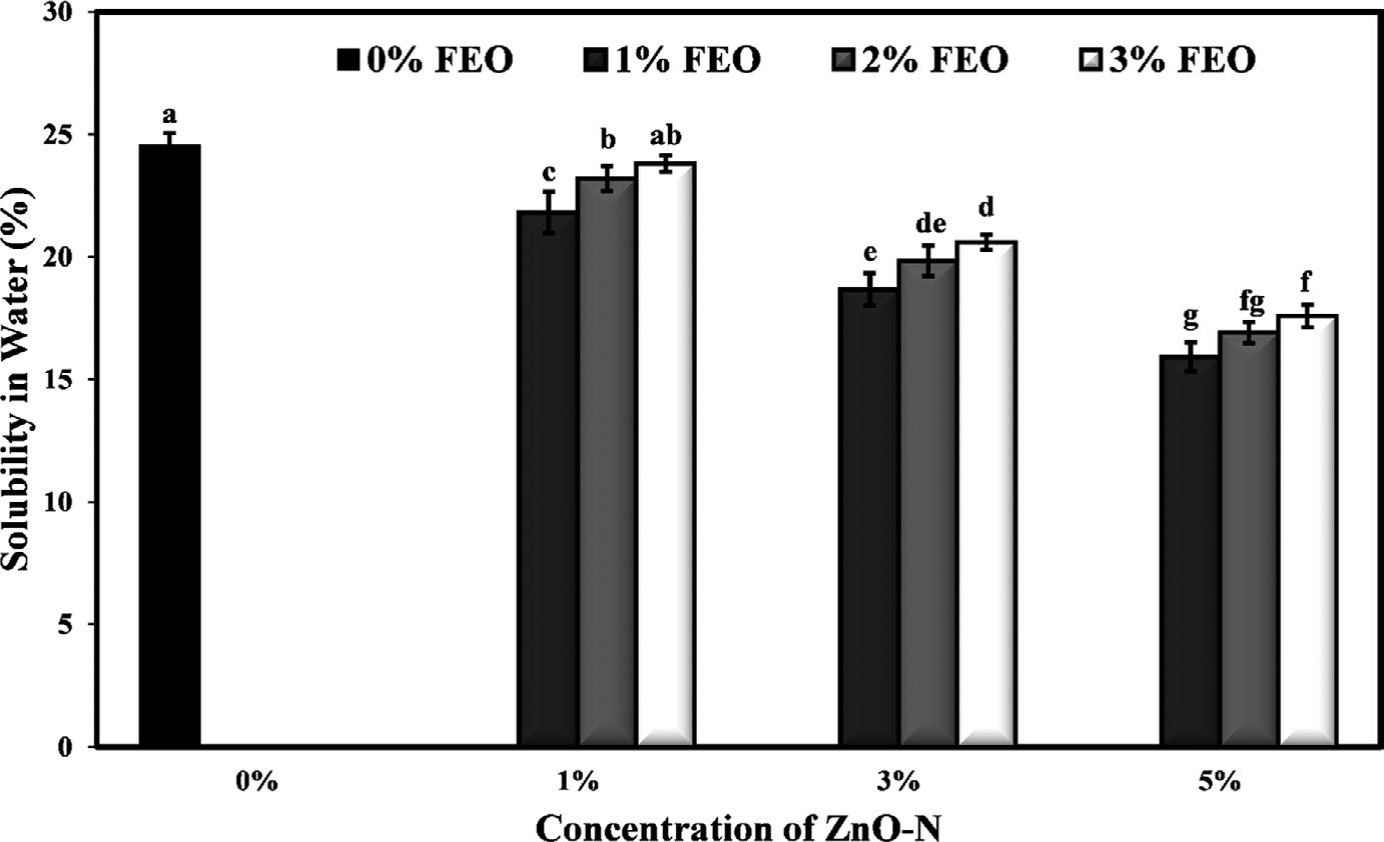
Effects of nano‐ZnO and fennel essential oil on water solubility of (%) potato starch films

The combined effect of ZnO nanoparticles and FEO on potato starch‐based films is given in Figure 2. The addition of different levels of ZnO nanoparticle and FEO to the bionanocomposite film samples resulted in a significant reduction in the solubility of the films in water (*p* < .05) so that the highest solubility was observed in control (24.54%). Increasing the concentration of ZnO‐N in the films caused a significant decrease in solubility (*p* < .05), while increasing the level of FEO has not effect on this parameter. Generally, the lowest solubility percentage was for the film sample containing a combination of 5% ZnO‐N and 1% FEO (15.92%). Decreased solubility of starch films due to the incorporation of different levels of ZnO nanoparticles can be attributed to the formation of strong hydrogen bonds between the starch network and the nanoparticles. The addition of ZnO nanoparticles to the polymer matrix reduces the hydroxyl groups available to water molecules and thus reduced the hydrophilicity of starch films. The increase in the starch film solubility by increasing FEO concentration is probably due to the penetration of phenolic compounds of the essential oil into the interior of the starch network and the reduction of intermolecular bonds.

The researchers showed that the solubility of cellulose films was significantly increased by the addition of *Scrophularia striata* extract (Sokhtezari et al., 2017). Jafarzadeh et al. (2017) stated that the addition of ZnO nanorods to the semolina nanocomposite reduced the solubility of the films in the water. The improving effect of nanoparticles on the stability of polymer films against water has been reported by different researchers (Ahmed et al., 2016).

### 3.3 Effect of ZnO‐N and FEO on water absorption capacity (WAC) of starch films

**FIGURE 3:**
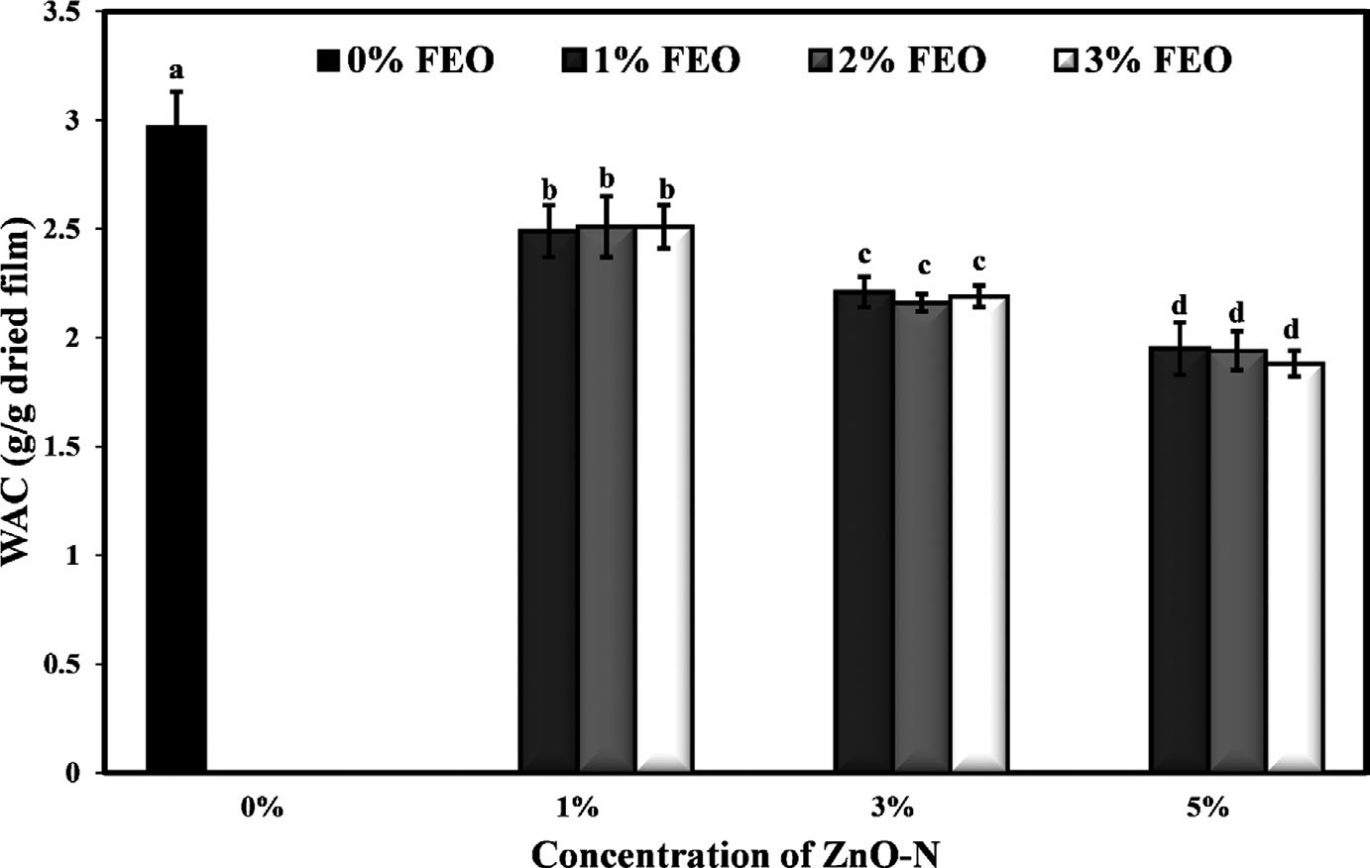
Effects of nano‐ZnO and fennel essential oil on water absorption capacity (g/g dried film) of potato starch films

In Figure 3, the effect of adding different combinations of ZnO‐N and FEO on the WAC values of potato starch bionanocomposite is shown. The results demonstrated that by incorporating the combination of different levels of ZnO‐N and FEO into the film samples, the values of WAC were significantly reduced (*p* < .05). Increasing the ZnO‐N in the film samples led to an increase in WAC (*p* < .05), but with increasing the concentration of FEO, no significant change in WAC was observed. The average values of water absorption capacity of different bionanocomposite films were in the range of 1.88–2.97 g water/g dried film.

When films made from proteins or carbohydrates absorb water, they lead to changes in their structure, so details about the water absorption properties of biopolymer films are essential depending on the type of application. Moisture absorption in films is due to the hydroxyl groups in biopolymers such as starches that bind to water. The addition of ZnO‐N to the starch matrix reduces the WAC amount of films by reducing the hydroxyl groups available to water molecules. The lack of significant effect of FEO on the WAC amount of starch films is probably because essential oils can reduce intermolecular bonds in the starch network and help absorb moisture by increasing free hydroxyl groups. On the other hand, the presence of phenolic and hydrophobic compounds in the essential oil prevents the film from absorbing too much water. Therefore, the incorporation of FEO did not significantly affect the water absorption capacity of films. Ahmadi et al. (2019) found that incorporating cellulose nanofiber and Miswak extract had no significant effect on the water absorption of cellulose films. Jafarian et al. (2020) observed that with increasing the level of ZnO nanoparticles in basil seed mucilage‐based films, WAC amounts decreased significantly. Similarly, Oleyaei et al. (2016) also reported an increase in the hydrophilicity and decrease in the WAC values of potato starch‐based films due to the addition of titanium dioxide nanoparticles.

### 3.4 Effect of ZnO‐N and FEO on water vapor permeability (WVP) of starch films

**FIGURE 4:**
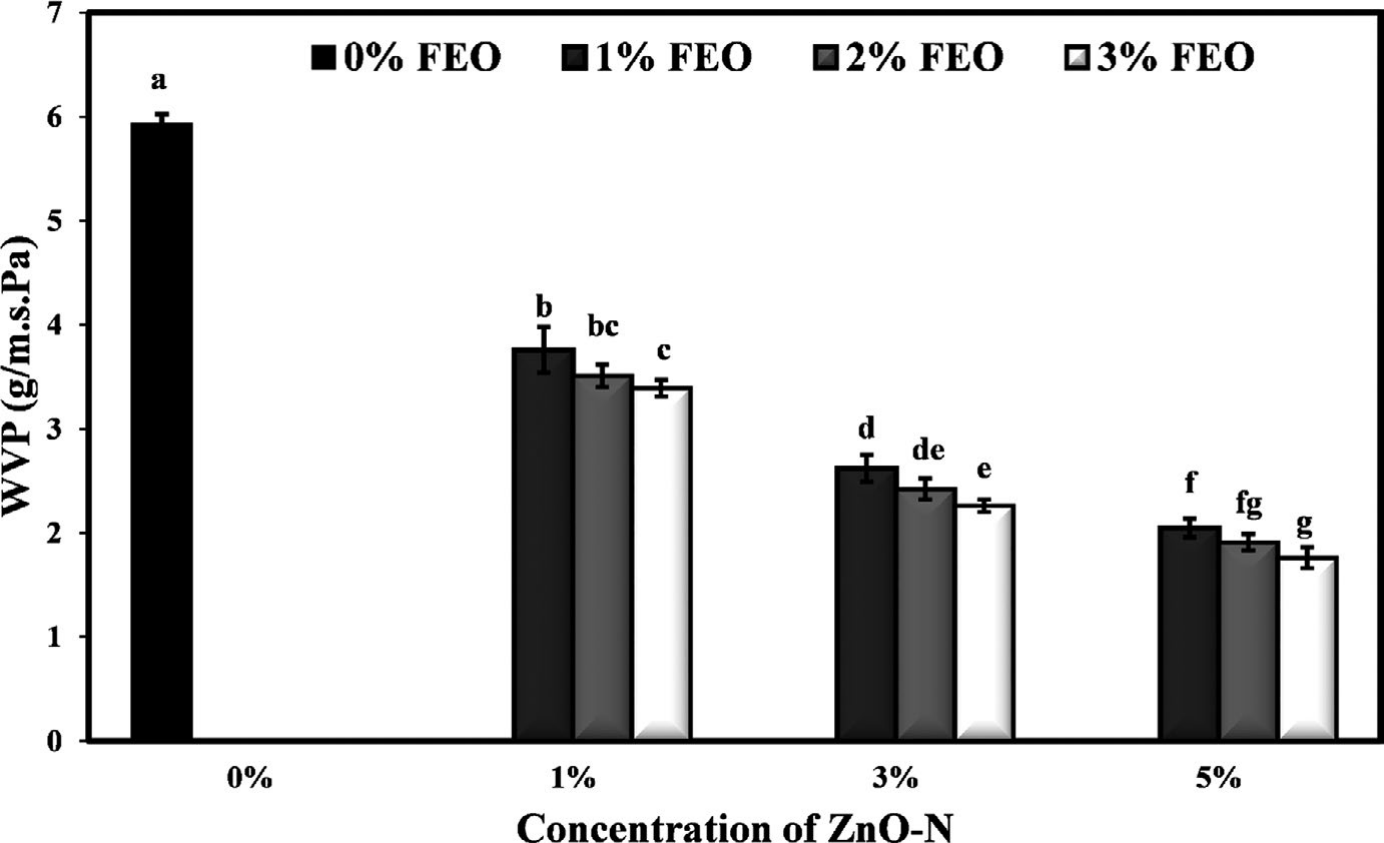
Effects of nano‐ZnO and fennel essential oil on vapor permeability (g/m.s. Pa) of potato starch films

Permeability is a process of solubility and diffusion in which water vapor penetrates from one side of the film and then diffuses to the other side of the film. Polysaccharide films, due to their hydrophilic nature, show mainly weak water vapor barriers (Aboulghasemi et al., 2012). In addition, glycerol used as a plasticizer in film production is a hydrophilic molecule that can be trapped between adjacent polymer chains, increasing molecular mobility and facilitating water vapor migration. Since the primary function of packaging in the food industry is to prevent or minimize moisture transfer between the food and the environment, water vapor permeability should be reduced as much as possible (Chiumarelli & Hubinger, 2014). The average values of WVP of films containing different ZnO‐N and FEO levels are compared in Figure 4. It shows that by incorporating different levels of nanoparticles and essential oil to starch films, WVP was significantly reduced (*p* <.05) so that the control showed the highest permeability (5.93 × 10^–10^ g/s.m. Pa). Increasing the concentration of ZnO‐N from 1% to 5% and FEO from 2% to 3% in starch films significantly reduced the WVP (*p* <.05). The lowest WVP amount was observed in the film containing the combination of 5% ZnO‐N and 3% FEO (1.76 × 10^–10^ g/s.m. Pa).

The addition of nanoparticles by dispersing in the film bed and compacting the film structure reduces the passage space of water molecules and makes their penetration path more difficult. Therefore, the penetrating molecules have to travel a long distance to cross the film, thus reducing the rate of transmission and penetration (Bourtoom, 2008; Müller et al., 2008). The decrease in WVP due to the addition of different levels of FEO is probably because water penetration in the film is done through their hydrophilic parts, while essential oils have a hydrophobic nature and can reduce the water vapor permeability by increasing the ratio of hydrophobic areas to hydrophilic (Hernandez, 1994). Farahnaky et al. (2014) reported that when the level of clay nanoparticles increased from 0% to 18%, WVP in gelatin films decreased significantly. Sokhtezari et al. (2017) also observed a significant increase in the WVP of active cellulose films due to the addition of the *Scrophularia striata* extract. Chahardehi Sirati et al. (2018) found a decrease in WVP of starch films due to the incorporation of ZnO nanoparticles. Similar results were reported by other researchers on the effect of nanoparticles on the WVP of nanocomposite films (De Moura et al., 2012).

### 3.5 Effect of ZnO‐N and FEO on oxygen permeability (OP) of starch films

**FIGURE 5:**
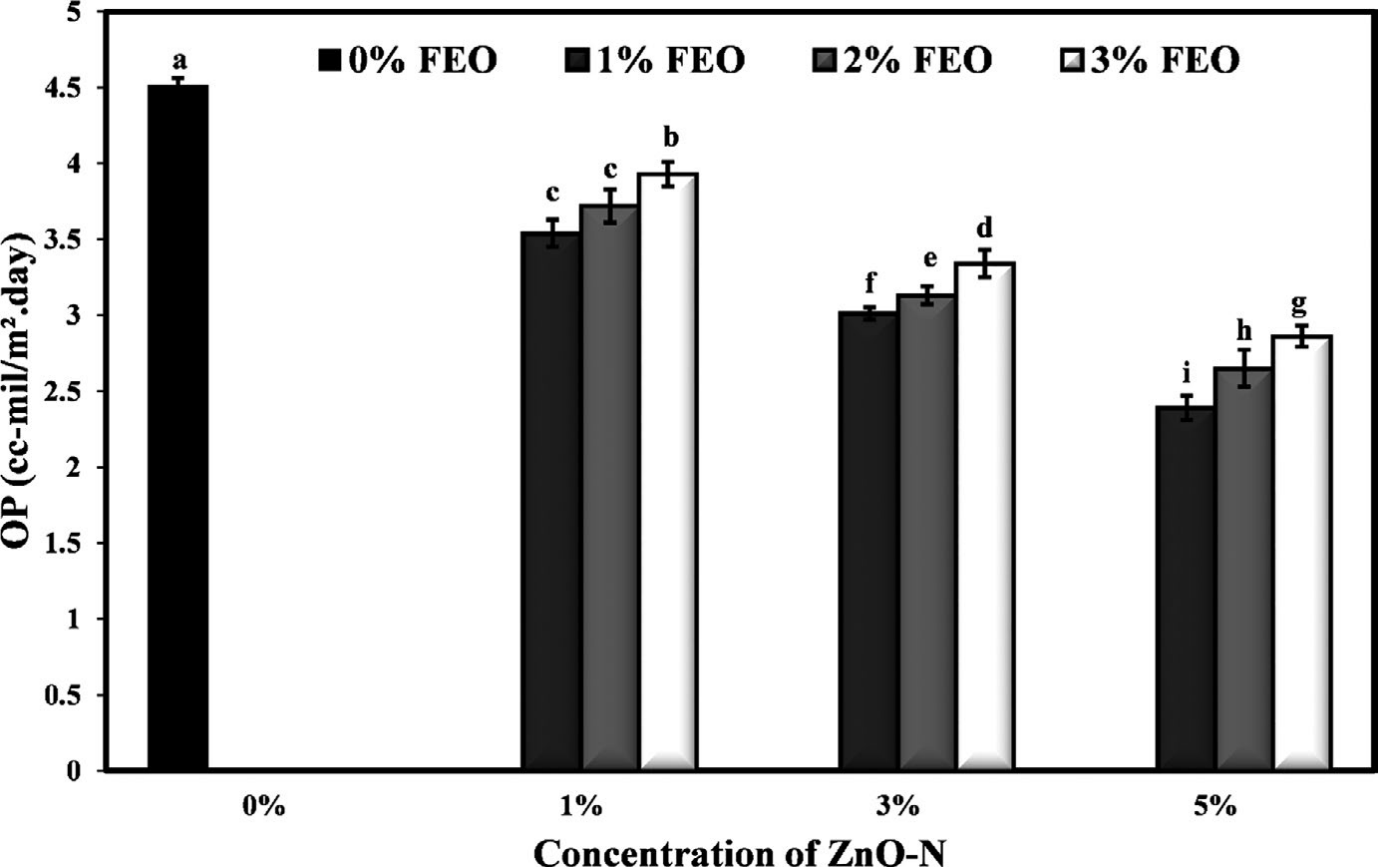
Effects of nano‐ZnO and fennel essential oil on oxygen permeability (cc‐mil/m^2^.day) of potato starch films

One of the important parameters for a food packaging material is its resistance to gasses. Excessive transfer of oxygen from the environment to the food inside the package may accelerate the oxidation process of lipids and reduce the nutritional value and quality of the food product (Wihodo & Moraru, 2013). The effect of combining different levels of ZnO‐N and FEO on oxygen permeability of potato starch films is presented in Figure 5. Incorporating different levels of nanoparticles and FEO into the films led to a significant reduction in the OP of samples (*p* <.05). By increasing the concentration of ZnO‐N in the films, the amount of permeability was significantly reduced, but increasing the levels of FEO led to an increase in the OP. In general, the highest amount of OP was related to the control (4.51 cc‐mil/m^2^.day), and the film containing 5% ZnO‐N and 1% FEO had the lowest amount (2.39 cc‐mil/m^2^.day).

Fillers such as nanoparticles can be considered as impermeable barriers to the movement of oxygen molecules, which reduce the oxygen permeability of packaging films by making the passageway difficult for oxygen molecules and gases (Zeppa et al., 2009). Increased OP due to the addition of FEO is probably related to the change in the structure of the film and the increase of cavities in them. Altiok et al. (2010) agreed that the incorporation of thyme essential oil in the chitosan films increased the permeability to oxygen. Jafarzadeh et al. (2017) observed a significant reduction in the oxygen permeability of semolina films due to the incorporation of ZnO nanorods. Other researchers have reported improved oxygen permeability of polymer films by the addition of nanoparticles (Nafchi et al., 2013; Teymourpour et al., 2015).

### 3.6 Effect of ZnO‐N and FEO on mechanical properties of starch films

Mechanical properties including tensile strength (TS), Young's modulus (YM), and elongation at break (EB) are among the most important parameters of food packaging films and are influenced by the type of raw material for film production and the environment around packaging (Abedinia et al., 2020). The mechanical properties of bionanocomposite films containing different concentrations of ZnO‐N and FEO are given in Table 1. As can be seen in this table, adding a combination of different levels of ZnO‐N and FEO to the starch films led to a significant increase in the values of TS and YM and reduced the amount of EB of the films (*p* <.05). The lowest values of TS (2.82 MPa) and YM (61.05 MPa) and the highest value of EB (27.64%) were obtained in the control sample. Increasing the level of ZnO‐N in the films increased the TS and YM of the samples (*p* < .05), while increasing the concentration of FEO increased the amount of EB significantly (*p* < .05). The film sample containing 5% ZnO‐N and 1% FEO had the highest TS (7.16 MPa) and YM (139.11 MPa) and the lowest TS (18.45%).

**TABLE 1:** Effects of nano‐ZnO and fennel essential oil on mechanical properties of potato starch films

Film samples	TS (MPa)	YM (MPa)	EB (%)
Control	2.82 ± 0.15 j	61.05 ± 0.27 j	27.64 ± 0.22 a
1% ZnO+1%FEO	4.20 ± 0.11 g	92.46 ± 0.35 g	25.39 ± 0.26 d
1% ZnO+2%FEO	3.89 ± 0.12 hr	91.78 ± 0.24 hr	25.83 ± 0.16 c
1% ZnO+3%FEO	3.61 ± 0.14 i	91.20 ± 0.19 i	26.41 ± 0.29 b
3% ZnO+1%FEO	5.54 ± 0.09 d	117.39 ± 0.34 d	22.78 ± 0.27 g
3% ZnO+2%FEO	5.35 ± 0.06 e	116.68 ± 0.26 e	23.23 ± 0.12 f
3% ZnO+3%FEO	5.11 ± 0.10 f	116.08 ± 0.23 f	23.62 ± 0.21 e
5% ZnO+1%FEO	7.16 ± 0.17 a	139.11 ± 0.25 a	18.45 ± 0.34 j
5% ZnO+2%FEO	6.72 ± 0.19 b	138.58 ± 0.19 b	18.99 ± 0.14 i
5% ZnO+3%FEO	6.35 ± 0.14 c	137.98 ± 0.29 c	19.35 ± 0.32 hr

Note

Values represent mean (*n* = 3) ± *SD*. Different letters in each column represent a significant difference at 5% level of probability among sample films.

Due to the fact that nanoparticles have a high specific surface area (surface to volume ratio), effective interactions between nanoparticles and the film base material are created. ZnO nanoparticles as a filler show a reinforcing and strengthening effect and thus increase the tensile strength and elastic modulus of nanocomposite films. The improving effects of nanoparticles on the strength of nanocomposite films have been confirmed by researchers (Chahardehi Sirati et al., 2018; Gholami et al., 2013; Reddy et al., 2018). Jebel and Almasi (2016) also demonstrated that the incorporation of ZnO nanoparticles into cellulose‐based films improved the tensile strength of samples. A reduction in the elongation percentage of the films due to the addition of nanoparticles was also observed by Hasheminya et al. (2018). However, fennel essential oil, due to its emollient role and interference in starch interactions, weakened the mechanical properties of the films. Similarly, Sokhtezari et al. (2017) reported that the addition of *Scrophularia striata* extract to active cellulose films reduced the TS and YM and increased the EB of the films. Moradi et al. (2012) also showed that the addition of grape seed extract to chitosan films reduced the strength of films.

### 3.7 Effect of ZnO‐N and FEO on color properties of starch films

The effect of a combination of ZnO‐N and FEO on color indexes of starch‐based bionanocomposite films is shown in Table 2. The highest L* (84.84) and lowest a* and b* indexes (−0.50 and 5.53, respectively) were observed in the control sample, by increasing the concentration of ZnO‐N and FEO in films led to a darker color of the films. The lowest amounts of color lightness (67.66) and the highest amounts of a* and b* indexes (4.13 and 15.85, respectively) were observed in films containing 5% ZnO‐N and 3% FEO. Jafarzadeh et al. (2017) also observed that the color of semolina‐based films darkened due to the addition of ZnO nanorods. Arfat et al. (2017) showed that the incorporation of Ag‐Cu nanoparticles to gelatin‐based film significantly reduced the color lightness and increased the a* and b* values. Darkening of the color of nanocomposite films due to the addition of nanoparticles has also been reported by other researchers (Kanmani & Rhim, 2014; Rhim et al., 2013; Zolfi et al., 2014). Gonçalves et al. (2020) also observed that due to the addition of sweet fennel essential oil to the cellulose acetate films, the amount of L* decreased, but a* and b* increased. Sun et al. (2020) also demonstrated a significant reduction in lightness intensity of konjac glucomannan/chitosan films due to the incorporation of nano‐ZnO and mulberry extract.

**TABLE 2:** Effects of nano‐ZnO and fennel essential oil on color indexes of potato starch films

Film samples	L*	a*	b*
Control	84.84 ± 0.82 a	−0.50 ± 0.14 j	5.53 ± 0.22 j
1% ZnO+1%FEO	81.25 ± 0.71 b	1.62 ± 0.12 i	7.31 ± 0.15 i
1% ZnO+2%FEO	80.08 ± 0.42 c	1.93 ± 0.18 hr	7.58 ± 0.09 hr
1% ZnO+3%FEO	79.01 ± 0.59 d	2.22 ± 0.09 g	7.93 ± 0.20 g
3% ZnO+1%FEO	76.41 ± 0.48 e	2.54 ± 0.17 f	12.40 ± 0.19 f
3% ZnO+2%FEO	75.28 ± 0.55 f	2.88 ± 0.14 e	12.78 ± 0.14 e
3% ZnO+3%FEO	73.98 ± 0.75 g	3.09 ± 0.04 d	13.15 ± 0.16 d
5% ZnO+1%FEO	70.42 ± 0.45 hr	3.58 ± 0.15 c	15.02 ± 0.17 c
5% ZnO+2%FEO	69.17 ± 0.54 i	3.84 ± 0.08 b	15.39 ± 0.15 b
5% ZnO+3%FEO	67.66 ± 0.62 j	4.13 ± 0.06 a	15.85 ± 0.21 a

Note

Values represent mean (*n* = 3) ± *SD*. Different letters in each column represent a significant difference at 5% level of probability among sample films.

### 3.8 Effect of ZnO‐N and FEO on antimicrobial activity of starch films

The antimicrobial activity of starch films containing a combination of ZnO‐N and FEO was investigated by the agar disk diffusion, and the obtained results are given in Table 3. The control film had no antimicrobial activity against *S. aureus*, *E. coli,* and *A. flavus,* and by adding and increasing the concentration of ZnO‐N and FEO in films, antimicrobial activity was increased, and the diameter of the inhibition zone also was increased significantly (*p* < .05). So that, the highest area of growth inhibition zone against *S. aureus* (146.15 mm^2^), *E. coli* (124.37 mm^2^), and *A. flavus* (104.88 mm^2^) was obtained in the films containing the highest level of ZnO‐N and FEO (5% ZnO‐N and 3% FEO). Generally, the antimicrobial activity of ZnO‐N was higher than FEO. The highest antimicrobial activity of bionanocomposite films was on gram‐positive bacteria, followed by gram‐negative and mold, respectively. The higher antimicrobial activity of essential oil against gram‐positive bacteria than gram‐negative bacteria is due to structural differences in the cytoplasmic membrane and cell wall of these bacteria. Because gram‐negative bacteria have an all‐encompassing cell membrane, it prevents the diffusion of hydrophobic compounds into the lipopolysaccharide coating (Oraki et al., 2011).

**TABLE 3:** Comparison of Inhabitation zone (mm^2^) of potato starch films containing nano‐ZnO and fennel essential oil

Film samples	*E. coli*	*S. aureus*	*A. flavus*
Control	0.00 ± 0.00 j	0.00 ± 0.00 j	0.00 ± 0.00 j
1% ZnO+1%FEO	33.77 ± 0.25 i	47.26 ± 0.21 i	27.28 ± 0.11 i
1% ZnO+2%FEO	35.96 ± 0.43 hr	48.90 ± 0.14 hr	28.84 ± 0.15 hr
1% ZnO+3%FEO	36.89 ± 0.55 g	49.70 ± 0.11 g	29.62 ± 0.13 g
3% ZnO+1%FEO	93.48 ± 0.27 f	108.27 ± 0.17 f	77.42 ± 0.19 f
3% ZnO+2%FEO	95.42 ± 0.31 e	109.94 ± 0.15 e	78.86 ± 0.11 e
3% ZnO+3%FEO	97.34 ± 0.35 d	111.02 ± 0.24 d	80.10 ± 0.09 d
5% ZnO+1%FEO	121.60 ± 0.29 c	142.15 ± 0.13 c	102.13 ± 0.07 c
5% ZnO+2%FEO	122.85 ± 0.41 b	144.03 ± 0.16 b	103.96 ± 0.13 b
5% ZnO+3%FEO	124.37 ± 0.26 a	146.15 ± 0.28 a	104.88 ± 0.15 a

Note

Values represent mean (*n* = 3) ± *SD*. Different letters in each column represent a significant difference at 5% level of probability among sample films.

**FIGURE 6:**
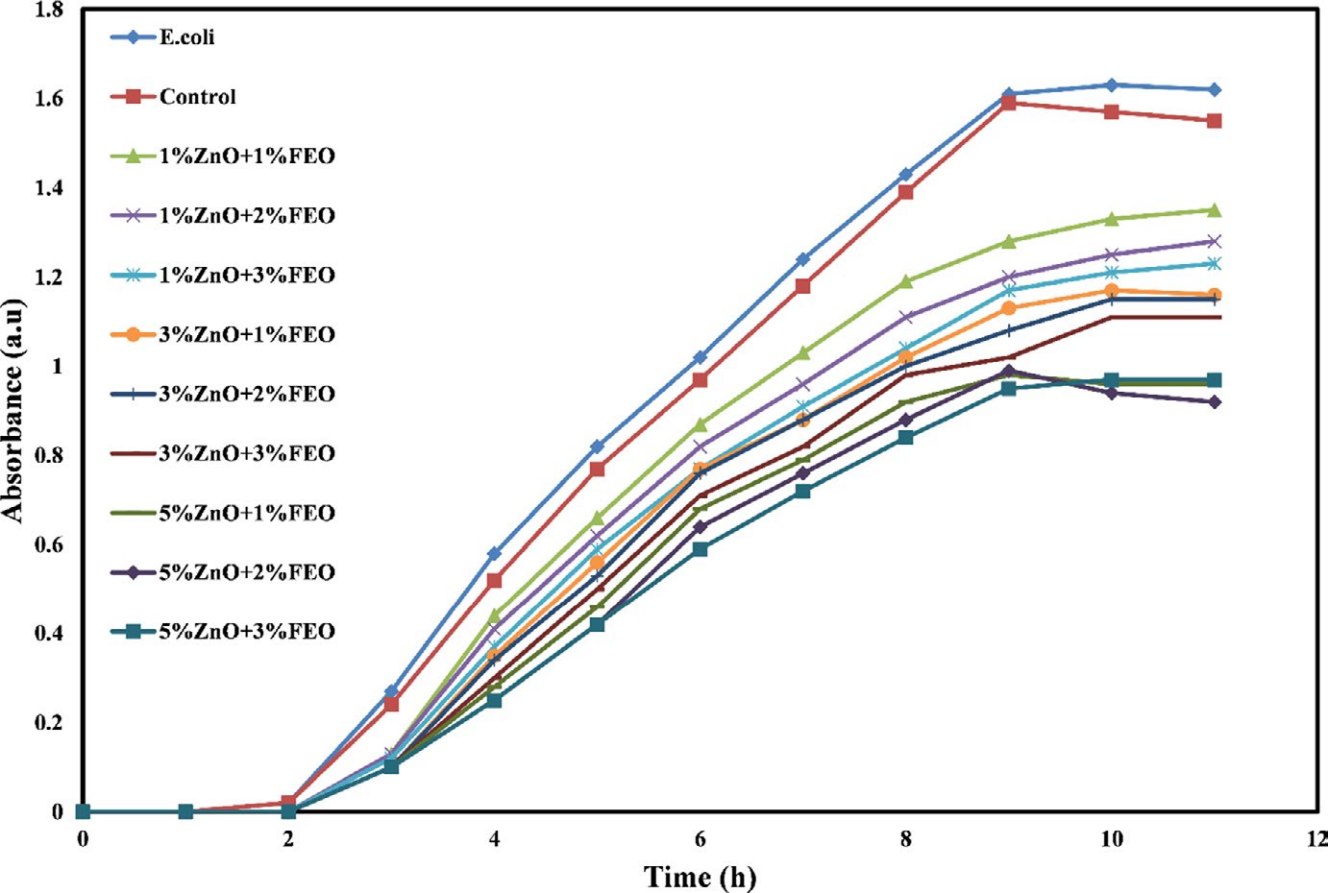
Microbial growth curve of *Escherichia coli* against potato starch films containing nano‐ZnO and fennel essential oil

**FIGURE 7:**
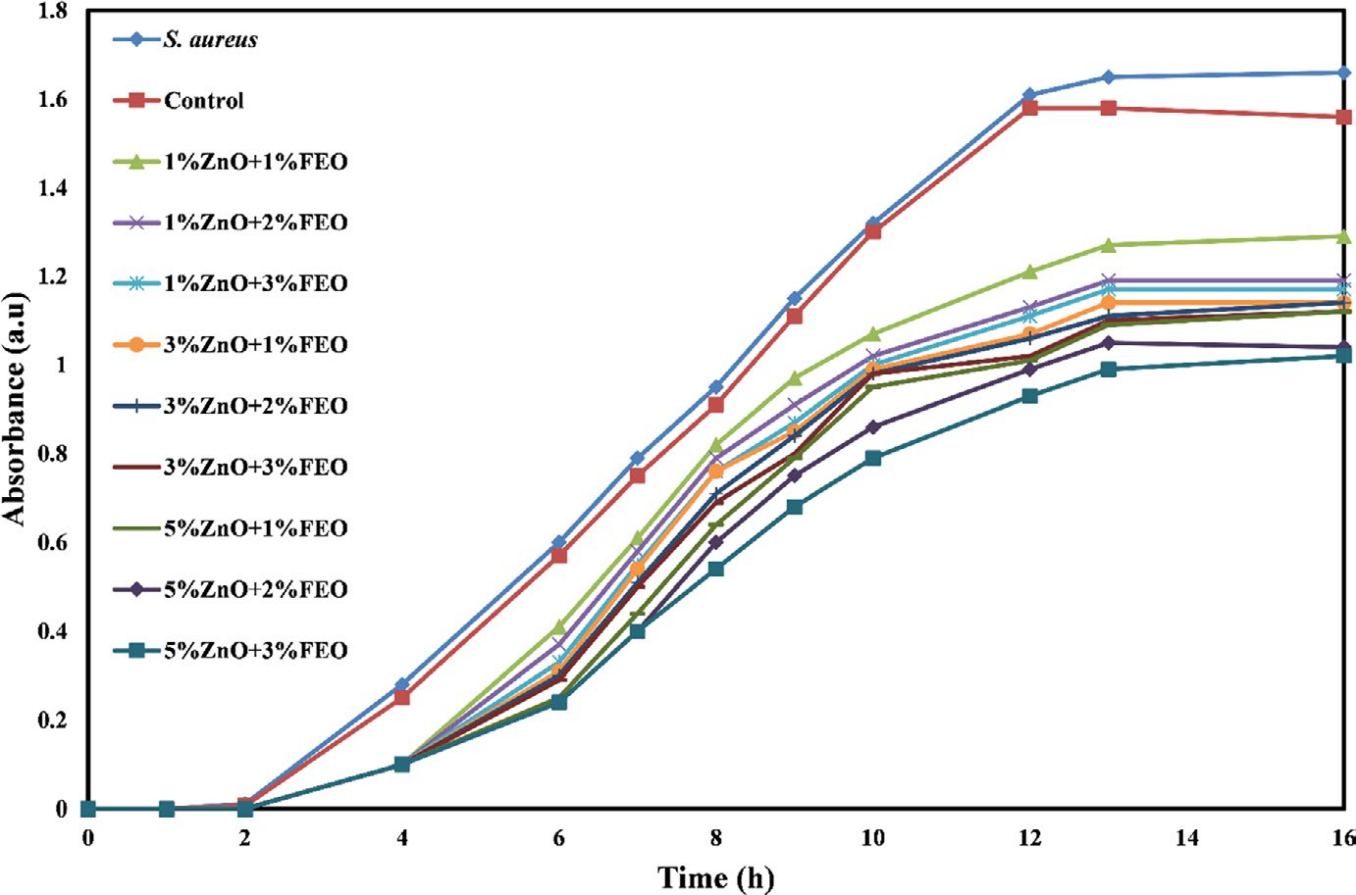
Microbial growth curve of *Staphylococcus aureus* against potato starch films containing nano‐ZnO and fennel essential oil

**FIGURE 8:**
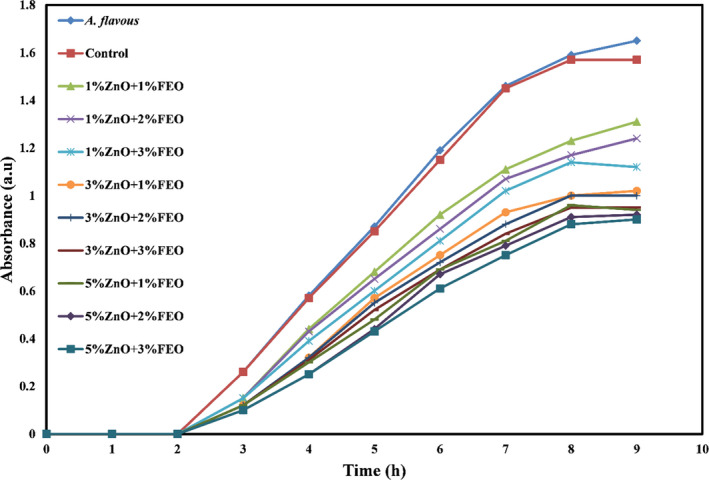
Microbial growth curve of *Aspergillus flavus* against potato starch films containing nano‐ZnO and fennel essential oil

Figures 6, 7, and 8 show the microbial growth curves of *S. aureus, E. coli,* and *A. flavus*, respectively. As expected, and as can be seen in these figures, the potato starch film had no antimicrobial activity, and by incorporating the combination of ZnO‐N and FEO into the starch films and increasing their concentrations, antimicrobial activity was significantly increased (*p* <.05) and the microbial growth curves shifted downward so that the lag phase increased and the log phase decreased. According to the microbial growth curves, the highest inhibition was related to the bionanocomposite films containing 5% ZnO‐N and 3% FEO.

Several mechanisms have been proposed for the antimicrobial action of metal nanoparticles, the most important of which include the catalytic activity of oxidation and reduction, which affects the active site of enzyme, DNA, and the activity of ribosomes and disrupts metabolic activity (Bruna et al., 2012). Induction of reactive oxygen species such as hydrogen peroxide, hydroxyl radicals, and superoxide (Emamifar et al., 2010) and cell wall damage are also suggested mechanisms for the antimicrobial activity of nanoparticles (Li et al., 2011).

One of the important properties of essential oil and its effective compounds is its hydrophobicity, which enables them to destroy the cell wall of bacteria or the wall of mitochondria and cause the destruction of the cellular structure and greater permeability of cells. Migration of ions and other cellular contents can also occur, which depletes the contents of the microbial cell and release their sensitive substances. The essential oils that have the strongest antimicrobial activity against food poisoning microorganisms contain high levels of active phenolic compounds usually. Hence, their mechanism of action seems to be similar to other phenolic compounds. These active compounds usually disrupt the function of the cell membrane and break and disrupt the proton kinetic force, electron current, and the active transfer and coagulation of cell contents. Essential oil compounds also affect proteins in the cytoplasmic membrane (Oraki et al., 2011). Researchers have stated that FEO has desirable antimicrobial activity, which is due to the presence of flavonoids, terpenoids, carotenoids, and coumarins in the essential oil of this plant (Singh et al., 2006). There are more than 30 types of terpene or terpenoid compounds in fennel essential oil (Guillén & Manzanos, 1996). Effective antimicrobial activity of cellulose acetate films containing sweet fennel essential oil against *S. aureus* and *E. coli* has been reported by Goncalves et al. (2020). Kadam et al. (2014) observed that the addition of TiO_2_ and SiO_2_ nanoparticles to films based on soy protein isolate and corn zein protein significantly increased the antimicrobial activity of these films. Gharavi Ahangar et al. (2015) also reported that the addition of ZnO nanoparticles to polyvinyl alcohol films significantly inhibited the growth of *E. coli* bacteria.

## 4 CONCLUSION

The results of this study demonstrated that the incorporation of ZnO‐N and FEO combination to potato starch‐based nanocomposite films significantly improved the tensile strength and barrier properties of starch films against oxygen and water vapor. Bionanocomposite films had higher moisture resistance than control films. However, they were darker in color. ZnO‐N and FEO showed significant antimicrobial activity in starch films so that by increasing the concentration of these additives, the antimicrobial activity of bionanocomposite films was improved significantly. In general, the best characteristics, as well as antimicrobial performance, were observed in the starch film containing 5% ZnO‐N and 3% FEO.

## CONFLICT OF INTEREST

The authors declare no conflict of interest.

## AUTHOR CONTRIBUTION

**Hamid Babapour:** Data curation (equal); Formal analysis (equal); Funding acquisition (equal); Investigation (equal); Writing‐original draft (equal). **Hossein Jalali:** Project administration (equal); Supervision (equal); Validation (equal); Visualization (equal). **Abdorreza Mohammadi Nafchi:** Conceptualization (equal); Methodology (equal); Project administration (equal); Supervision (equal); Writing‐review & editing (equal).

## ETHICAL APPROVAL

This study does not involve any human or animal testing.

## Data Availability

The data that support the findings of this study are available from the corresponding author, upon reasonable request.
